# Methane mitigation potentials and related costs of China's coal mines

**DOI:** 10.1016/j.fmre.2023.09.012

**Published:** 2023-12-29

**Authors:** Yating Kang, Peipei Tian, Jiashuo Li, Hetong Wang, Kuishuang Feng

**Affiliations:** a Institute of Blue and Green Development, Shandong University, Weihai 264209, China; b Department of Geographical Sciences, University of Maryland, College Park, MD 20742, USA

**Keywords:** Methane, Coal mine, Mitigation potential, Economic cost, China

## Abstract

Mitigating methane (CH_4_) emissions from China's coal mines as the largest contributor to anthropogenic CH_4_ emissions is vital for limiting global warming. However, the knowledge about CH_4_ mitigation potentials and economic costs of Chinese coal mines remain poorly understood, which hinders the formulation of tailored CH_4_ mitigation strategies. Here, we estimate and project China's provincial coal mine methane (CMM) emissions, mitigation potentials and costs under various coal production scenarios, by integrating the dynamic emission factors of CMM and key abatement technologies. We find that through continuous coal cuts and available CMM mitigation measures, China's CMM emissions can be reduced by 65%-78% (10.9 Tg-13.1 Tg) in 2060, compared with the 2021 level. CH_4_ emissions from abandoned coal mines will far exceed those from coal mining under the 2060 carbon-neutral scenario, especially in northeastern China. It was also found that CMM mitigation is not economically feasible at present, but may be the most cost-effective solution as CO_2_ prices increase. All coal-producing provinces can achieve CMM mitigation below 50 RMB/t CO_2_e in 2060. Inner Mongolia is identified as a hotspot for CMM mitigation with huge potential and lower cost. Our prospective assessment can provide insights into China's CMM mitigation in response to climate change.

## Graphical abstract

**Figure:**
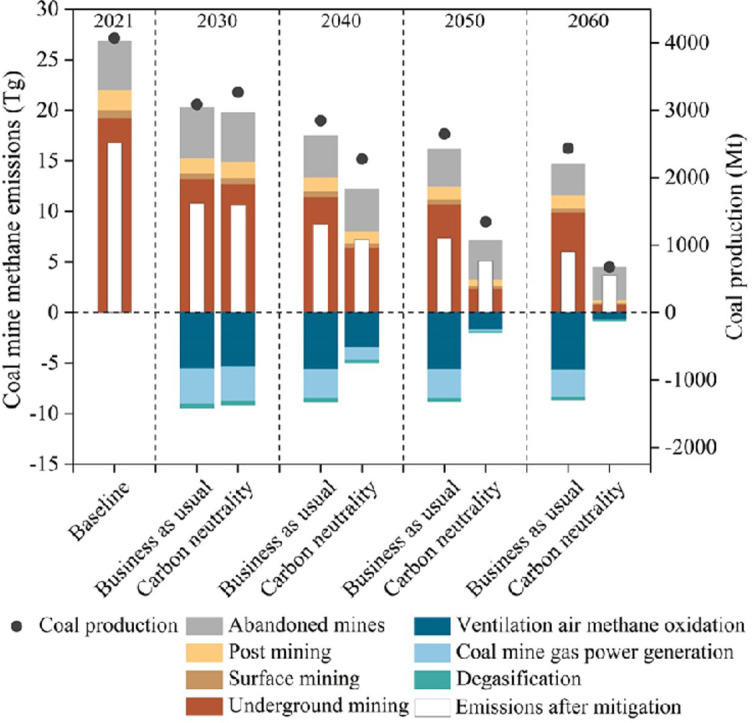

## 1 Introduction

Non-CO_2_ greenhouse gas (GHG) cuts can greatly help ease the burden of carbon abatement toward the Paris Agreement [1,2]. Mitigating methane (CH_4_), the most important non-CO_2_ GHG with a short atmospheric lifetime and high heat-trapping potential, is the most effective way to tackle near- and medium-term climate warming [3]. China's 14th Five-Year Plan (2021–2025) underscores the importance and urgency of deep CH_4_ mitigation [4]. In China, the world's top coal producer, coal mines are the largest source of anthropogenic CH_4_ emissions. Coal mine methane (CMM) contributes to over one-third of the national total CH_4_ emissions [5], [6], [7]. CMM emissions are a major hurdle for China's CH_4_ removal response.

According to the annual report on coal industry development [8], there were approximately 4,500 coal mines in operation scattered across 26 provinces in 2021 with various coal ranks, reserves, and geological conditions [9]. In the coming decades, a stringent carbon neutrality pledge will drive China's unprecedented transition away from coal. This means the phaseout of upstream coal mines [10]. The diversity and large number of coal mines and uncertainty of the phaseout pathway make it a challenge to formulate refined CMM mitigation policies. Additionally, CMM mitigation technologies have not been widely deployed in China, where economic activities, geological conditions, and applicable mitigation technologies vary largely between provinces [11]. Therefore, to guide tailored mitigation, it is imperative to evaluate and project China's CMM mitigation potentials and related costs under various coal mine phaseout scenarios at the provincial level.

An accurate accounting of total CMM emissions from coal mining, post mining and abandoned coal mines is a prerequisite for effective mitigation. Several studies have reported China's CMM emissions, with varied results and less consideration of abandoned coal mines [12], [13], [14], [15]. Importantly, once coal production is halted and the mine is abandoned, it continues to release CH_4_ for a long time [16,17]. The closure of specific coal mines in a province also affects in-situ CH_4_ content, which further results in year-to-year variability in the local emission factors (EFs) of coal mining [15]. A recent study by Gao et al. [15] incorporated abandoned coal mines for CMM emissions accounting in China; however, there is still a lack of forecasting with the latest data. Some studies that project future CMM emissions are based on constant national-level EFs with large uncertainties [18,19]. To improve the accuracy of the results, a comprehensive evaluation of CMM emissions must fully consider abandoned coal mines and the dynamic change in EFs.

CMM mitigation potential projections incorporating the diversity of climate change paths and regional differences could provide a foundation for refined CH_4_ mitigation planning. A global-scale study used the Greenhouse Gas and Air Pollution Interactions and Synergies (GAINS) model to assess CMM emissions and deployment of mitigation technologies in 2030 for 83 countries/regions [20]. Based on a top-down integrated model, Lin et al. [19] predicted total CMM emission peaks in China after the adoption of mitigation technologies for coal mining. However, this estimate is based on country-level parameters, which are too coarse to reflect the spatial heterogeneity of coal mines in China. Many researchers have also conducted inverse modeling (top-down) analysis of CMM observations [21,22]. However, the lack of prior information (i.e., spatial distribution of emission) may lead to biased estimation. As bottom-up analysis reveals plant-level information, it can capture changes in regional emissions [13]. Owing to the significant heterogeneity of China's coal mines, the phaseout of individual coal mines in the context of carbon neutrality necessitates a better bottom-up forecast. The aggregated CMM mitigation potentials at the point source level also need to be explored, quantified and better understood.

Looking beyond emissions and mitigation potentials, economic cost is also an important factor that will determine the implementation of CMM mitigation measures. The marginal abatement cost curves of CH_4_ estimated by Teng et al. [18] suggested that by introducing a reasonable level GHG tax (20 RMB/t CO_2_-equivalent), a 42.5% emission reduction can be achieved by 2050. Coal mine CH_4_ mitigation cost is related to the utilization mode and concentration of CH_4_. Currently, high CH_4_-content coal mine power generation technology, which offers a relatively low cost, has reached a mature stage. However, the cost of ventilation air methane oxidation technology for low-concentration CH_4_ is high, and only a few demonstration projects exist [23]. Yang et al. [24] found that China's coalbed methane clean development mechanism (CDM) projects can reduce the average mitigation cost of CMM to 27.5 RMB/t CO_2_e. Considering the cost reduction of accumulated experience, investigation of the economic impacts of large-scale deployment of CMM mitigation technologies in China is still scarce.

Here, we explore China's provincial CMM emissions, mitigation potentials, and related costs by combining the dynamic EFs of CMM, various CMM mitigation technologies, and long-term scenario analysis. Specifically, future national coal production is simulated by a bottom-up model, the Global Change Analysis Model (GCAM). National production is further spatially downscaled for each province using linear regression forecasts. We derive provincial dynamic EFs of CMM based on the latest point-source coal mine data and coal mine retirement strategies. In addition, we project the CMM emission reductions and costs of the large-scale deployment of key CMM mitigation technologies by 2060. Our study provides not only a useful tool to project CMM emissions but also insights for formulating appropriate CH_4_ mitigation roadmaps.

## 2 Methods

**Fig. 1:**
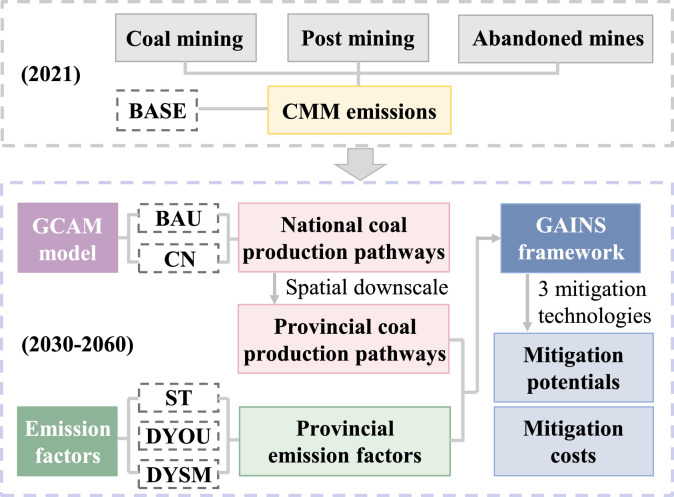
**Methodological framework of this study.** An integrated assessment framework is used to investigate China's coal mine methane (CMM) mitigation potentials and costs. CMM emissions from coal mining, post mining and abandoned mines are first calculated in a 2021 baseline (BASE) year. Future scenarios of coal production are based on different policy assumptions, i.e., business as usual (BAU) and carbon neutrality (CN). The Global Change Analysis Model (GCAM) is used to simulate national coal production pathways, which are further downscaled for each province. Three types of provincial emission factors of CMM are applied, namely, static emission factors (ST), dynamic emission factors with strategies of early retirement of outburst coal mines (DYOU), and dynamic emission factors with strategies of early retirement of small coal mines (DYSM). Following the Greenhouse Gas and Air Pollution Interactions and Synergies (GAINS) model framework, CMM mitigation potentials and costs from 2030 to 2060 are finally quantified under different scenarios pairing coal production pathways and emission factors.

This study, which is a comprehensive assessment of CMM mitigation potential and cost in China, applies an integrated assessment framework (Fig. 1).

### 2.1 Coal mine methane emissions accounting

Following the inventory guidelines of the Intergovernmental Panel on Climate Change (IPCC), CMM emissions calculated using activity data (i.e., coal production) and emission factors are defined by Eq. 1 [25]. Depending on the data availability, the Tier 2 method based on country- or basin-specific EFs is adopted for underground mining. The Tier 1 method based on the global average EFs is used for surface mining, post underground and surface mining, and abandoned coal mines:

(1) CMM=CP×EF

where *CMM* is the coal mine methane, *CP* is the coal production, and *EF* is the emission factor.

#### 2.1.1 Activity data collection for the baseline year

The total coal production for each province (including underground and surface mining) in 2021 was obtained from the National Bureau of Statistics of China. Because surface mining data are not available in official inventories, previous studies have commonly used the national average proportion of surface mining (5%) to estimate the corresponding production. In this study, we collect the latest data on the amount of coal production from surface mines to calculate its proportion (Table S3 in Supplementary Material), which is assumed to be constant by 2060. Coal production from underground mining is the difference between total production and production from surface mining. The cumulative number of China's provincial abandoned coal mines between 2010 and 2019 is from a recent study [15], and the numbers for 2020 and 2021 are collected from provincial government announcements. The aggregated number of abandoned coal mines before 2021 was then checked against the National Energy Administration's announcement on the number of coal mines in production. The results of the check showed that China's total number of abandoned coal mines reached approximately 12,800 by 2021 (Table S4).

#### 2.1.2 Coal production forecasting model

Combining the integrated assessment model GCAM and the downscaling method, we develop a forecast modeling framework for China's provincial coal production from 2030 to 2060.

GCAM (https://github.com/JGCRI/gcam-core/releases) is a market equilibrium model that represents key interactions between the economic, energy, land, and climate systems in 32 geopolitical regions in the world (China is divided separately). It solves the market prices and quantities of various energy, agricultural and GHG markets, which are driven by exogenous assumptions about population and labor productivity.

In this study, GCAM 5.4 was used to simulate the long-term evolution of China's coal supply under complex system interactions. Specifically, the energy module of GCAM starts from primary energy (i.e., coal and other fossil fuels) production, goes through energy transformation and distribution, and is finally used for end-use energy demands (in buildings, transportation, and industrial sectors) and final energy commodities (e.g., electricity, diesel). Primary energy production is determined by an exogenous supply curve that stipulates the availability of energy production as a function of energy prices. Primary energy is traded globally, and its future price changes originate from the equilibrium price of the entire energy demand in the GCAM. The coal supply curve is based on the number of resources (EJ), from which coal production in different periods can be obtained.

Future national coal production is further downscaled using linear regression forecasting of coal production for each province. To provide a reasonable initial forecast, we collect historical provincial coal production data over the past decade from the China Energy Statistical Yearbook [26], and then preprocess these raw data through the weighted moving average (WMA) method to better fit the production trend (details in Supplementary Material) [12]. The final projections of provincial coal production (*y^’^_i,j_*) are calculated according to Eq. 2:

(2) $$y_{i,j}^{\prime }=y_{i,j}-y_{i.j}\times \frac{Y_{2021}}{Y_{j}}$$

where *y_i,j_* is the weighted average coal production of province *i* for year *j*, Y_2021_ and *Y_j_* are the national coal production for year 2021 and year *i*, respectively.

#### 2.1.3 Emission factors derivation

We derive the production weighted EFs of underground mining at the provincial level using the matrix product of static EFs and the coal production proportions of the two mine types. Because of the similarities in local practices and coal mine basins, static EFs of low and high CH_4_-content coal mining for a given province are assumed to follow the same probability distribution, which is based on a recent study by Gao et al. [15]. For future provincial production of the two categories of coal mines, we assume that the operating coal mines would run at their existing capacity until their resources are exhausted or they are shut down. The production proportions of the remaining two mine types are calculated based on the reduced production capacity under the constraints of provincial total coal production from 2030 to 2060. Through the matrix operation, we obtain the provincial dynamic EFs of underground mining considering the increasing number of abandoned mines during the forecasting period (Table S5).

EFs of post underground mining, surface mining, and post surface mining are the default values recommended by IPCC, which are 0.94 m^3^/t, 1.2 m^3^/t, and 0.1 m^3^/t, respectively. According to the 2019 refinement of the 2006 IPCC Guidelines [27], the default EFs for abandoned coal mines vary slightly with the time interval between the mine closure year and the inventory year, as presented in Table S6.

### 2.2 Coal mine methane mitigation potential and cost estimation

CMM mitigation technologies have not yet been deployed on a large scale in China, but according to the coal industry report [8], the ratio of CMM utilization reached 50% in 2021. We use this ratio to estimate the provincial amount of CMM utilization (i.e., CMM emission reduction) in the baseline year, which is further subtracted from the total CMM emissions.

The CMM technical mitigation potential by 2060 is estimated using Eq. 3:

(3) $$MP_{i,j}=A_{i,j}\times \sum_{t}APP_{i,j,t}\times RE_{i,j,t}$$

where *MP_i,j_* is the CMM mitigation potential of province *i* for year *j; A_i,j_* is the projected coal production of province *i* for year *j; APP_i,j,t_* is the application rate of technology *t*, which depends on the proportion of the three mine types; *RE_i,j,t_* is the removal efficiency of technology *t* (Table 1).

**Table 1:** **Key parameters of CMM mitigation technologies**.

	Degasification	Ventilation air methane oxidation	Coal mine gas power generation
Applicable coal mine^a^	Surface mine	Low CH_4_-content underground mine	High CH_4_-content underground mine
Removal efficiency [28]	0.36	0.588	0.735
Unit cost^b^ (RMB/t coal)	0.539	6.087	5.668

a The application rates of the three technologies depend on the production proportions of the corresponding mine types [18], [20].

b The unit cost of ventilation air methane oxidation and coal mine gas power generation are derived from a demonstration project in China [24]. Owing to the lack of localized parameters, the unit cost of degasification is the global-average value from the GAINS model.

Following the widely used methodology framework of the GAINS model for calculating mitigation costs, we estimate the average mitigation cost of CMM technical mitigation to measure cost-effectiveness:

(4) $$C_{t}=I_{t}\left[\frac{{\left(1+r\right)}^{T_{t}}\times \;r}{{\left(1+r\right)}^{T_{t}}-1}\right]+M_{t}$$

(5) $$TC_{i,j}=A_{i,j}\times \sum_{t}APP_{i,j,t}\times C_{t}$$

(6) $$AC_{i,j}=\frac{TC_{i,j}}{MP_{i,j}}$$

where *C_t_* is the unit mitigation cost of technology *t*, RMB/t coal; *I_t_* is the upfront investment cost of technology *t*; $\left[\frac{{\left(1+r\right)}^{T_{t}}\times \;r}{{\left(1+r\right)}^{T_{t}}-1}\right]$ is the factor that annualizes the investment costs using discount rate *r* (5%) and technology-specific lifetime of *T_t_* years (20 years); *TC_i,j_* is the total CMM mitigation cost of province *i* for year *j; AC_i,j_* is the average mitigation cost of province *i* for year *j*, RMB/t CH_4_.

Considering the effects of technological change on mitigation costs and removal efficiency, the key parameters of CMM mitigation technologies in the long term are further adjusted according to the report of the U.S. Environmental Protection Agency [28]. We assume a 1% annual improvement in removal efficiency for nascent technologies but opted for a more conservative 0.5% annual improvement for mature technologies. The cost factors of CMM mitigation technologies in the USEPA report are used in this study to estimate the change in the unit cost of CMM mitigation technologies (Table S7). Accumulated experience through learning curves would lead to cost reduction.

### 2.3 Scenario analysis

In this study, we devise and simulate six scenarios by combining two coal production pathways with three groups of EFs of CMM (Table 2). The two coal production pathways are obtained by recursive calculation of the GCAM model under two climate policy assumptions, following the “middle of the road” shared socioeconomic pathway 2 (SSP2). One assumption is the business as usual (BAU), that is, the existing climate policies remain unchanged after 2021. The other is China's carbon neutrality target (CN), which features a linear decline in CO_2_ emissions from 12.77 Mt in 2021 to net zero in 2060 based on the near-term to net-zero approach [29,30]. In addition, considering that the EFs of CMM would change under various coal mine retirement strategies, we further devise three emission factor scenarios, namely, static emission factors (ST) scenario, dynamic EFs with strategies of early retirement of outburst coal mines (DYOU) scenario, and dynamic EFs with strategies of early retirement of small coal mines (DYSM) scenario. The ST scenario uses the EFs observed in the baseline year 2021 and assumes that the EFs will remain unchanged after 2021 [15]. Outburst coal mines are susceptible to coal fragmentation and gas outbursts, which pose safety risks. Small coal mines produce less than 0.3 Mt of coal annually, and their early retirement is in line with China's current strategy of “developing large units and suppressing small ones”.

**Table 2:** **Summary of scenarios in this study**.

Scenarios	Coal production pathways	Emission factors	Coal mine retirement strategies
BAU-ST	Business as usual	Static	–
BAU-DYSM	Business as usual	Dynamic	Small coal mines retire first
BAU-DYOU	Business as usual	Dynamic	Outburst coal mines retire first
CN-ST	Carbon neutrality	Static	–
CN-DYSM	Carbon neutrality	Dynamic	Small coal mines retire first
CN-DYOU	Carbon neutrality	Dynamic	Outburst coal mines retire first

Note: The ST emission factor scenario does not involve coal mine retirement, and the future emission factors of CMM in this scenario are 2021 baseline values.

### 2.4 Data sources

The original data of point-source coal mines are collected from the website of the Professional Knowledge Service System for Energy [5]. Enterprises reported the location, remaining reserves, coal gas level, production capacity in 2021, and other information on coal mines (Fig. S1). These data cover 78% of all coal mines and 82% of national coal production of the latest announcement by the National Energy Administration [31], which could well represent the actual operation situation of coal mines across the country.

## 3 Results

### 3.1 Coal mine methane emissions and mitigation potentials

#### 3.1.1 National coal mine methane emissions and mitigation potentials from 2021 to 2060

**Fig. 2:**
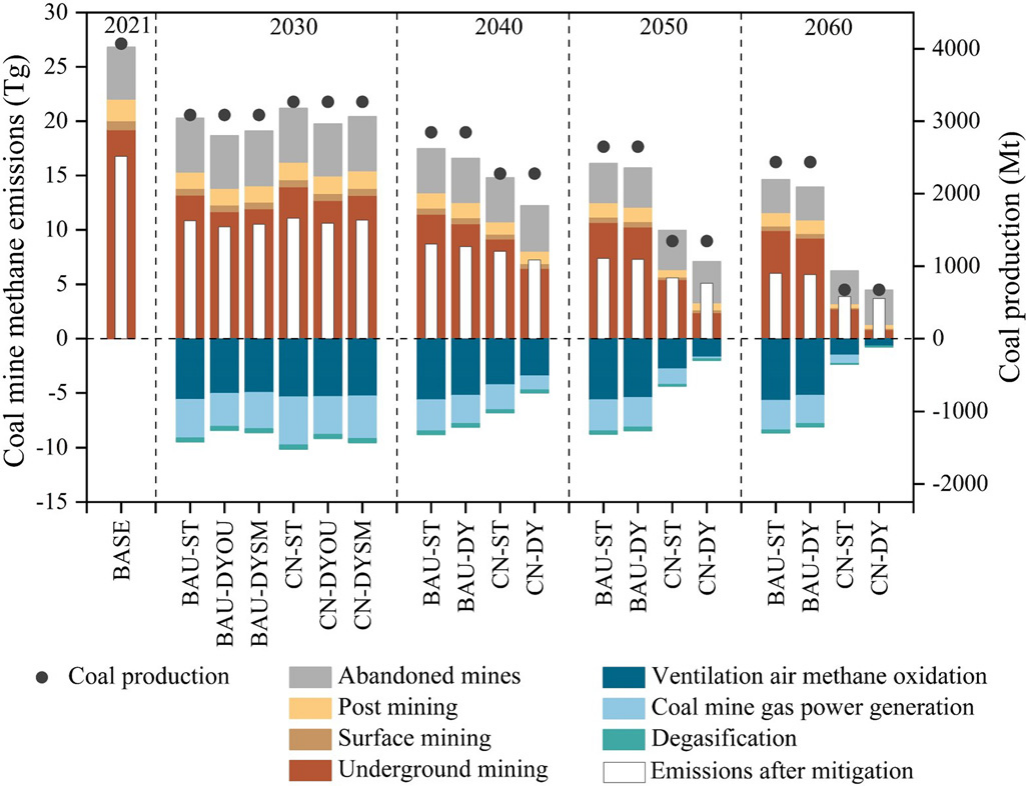
**China's CMM emissions and mitigation potentials from 2021 to 2060.** The colored bars on the positive axis of stacked histograms represent CMM emissions from four sources, and values on the negative axis represent mitigation potentials of three technologies. The total CMM emissions minus the mitigation potentials are the final emissions (i.e., Emissions after mitigation). BASE, baseline; BAU, business as usual; CN, carbon neutrality; ST, static; DYOU, dynamic emission factors with strategies of early retirement for outburst coal mines; DYSM, dynamic emission factors with strategies of early retirement for small coal mines. Details of these scenarios are provided in Table 2. All outburst and small coal mines would retire between 2021 and 2040 in our analysis, so there would only be four scenarios pairing two coal production pathways and two types of emission factors during the period 2040–2060.

China's total CMM emissions from underground and surface mining, post mining and abandoned mines were 26.81 Tg in the baseline year 2021 (Fig. 2). As half of the CH_4_ emissions from coal mining were utilized in 2021 [32], the rest are fugitive CMM emissions with an amount of 16.78 Tg. Abandoned coal mines were the second largest CMM source. CMM emissions from abandoned coal mines reached 4.75 Tg in 2021, and this figure is consistent with the latest study (4.7 ± 0.94 Mt) [17]. Moreover, the baseline estimate is in line with the estimation of the International Energy Agency's latest report on Chinese CMM emissions, which is comparable to the total anthropogenic CH_4_ emissions from Brazil (the fifth largest anthropogenic CH_4_ emission emitter) [33].

Future CMM emissions and mitigation potentials will follow the trajectory of coal production in different scenarios by 2060. CMM emission reductions come from coal mine phaseout and available CMM mitigation technologies. Specifically, to reach a carbon peak in 2030, the coal production in the CN scenario (3269 Mt) will be slightly higher than the BAU scenario (3087 Mt), resulting in larger CMM emissions. Under the same coal production constraint, the total CMM emissions of shutting down outburst coal mines first in 2030 (CN-DYOU) are 19.77 Tg, 0.66 Tg less than in CN-DYSM scenario (shutting down small coal mines first). Emission projections based on the constant EF of the CMM (CN-ST scenario) are larger than those of the other two scenarios. If three key mitigation technologies are deployed on a large scale, the CMM mitigation potential will reach 9.53 Tg in 2030 under the CN-DYSM scenario. This is larger than the mitigation potential of the CN-DYOU scenario, in which outburst coal mines are shut down first. However, the remaining CMM emissions (“Emissions after mitigation” in Fig. 2) of the CN-DYOU scenario will be lower (10.61 Tg), mainly due to the lower CH_4_ emissions from small coal mines that remain in operation. This indicates that early retirement strategies for outburst coal mines yield greater mitigation benefits. Moreover, the emissions could fall by 35%−39% from 2021 levels by 2030, which is higher than the overall CH_4_ emission reduction target proposed by the IPCC (20%−30%) [34]. After 2030, the accelerating reduction in coal consumption on the demand side will lead to a rapid decline in coal production toward a carbon neutrality future. All outburst and small coal mines will have retired by 2040. In the CN-DY scenario, the CMM emissions will decrease to 4.46 Tg in 2060, one-third of the emissions in the BAU-ST scenario.

**Fig. 3:**
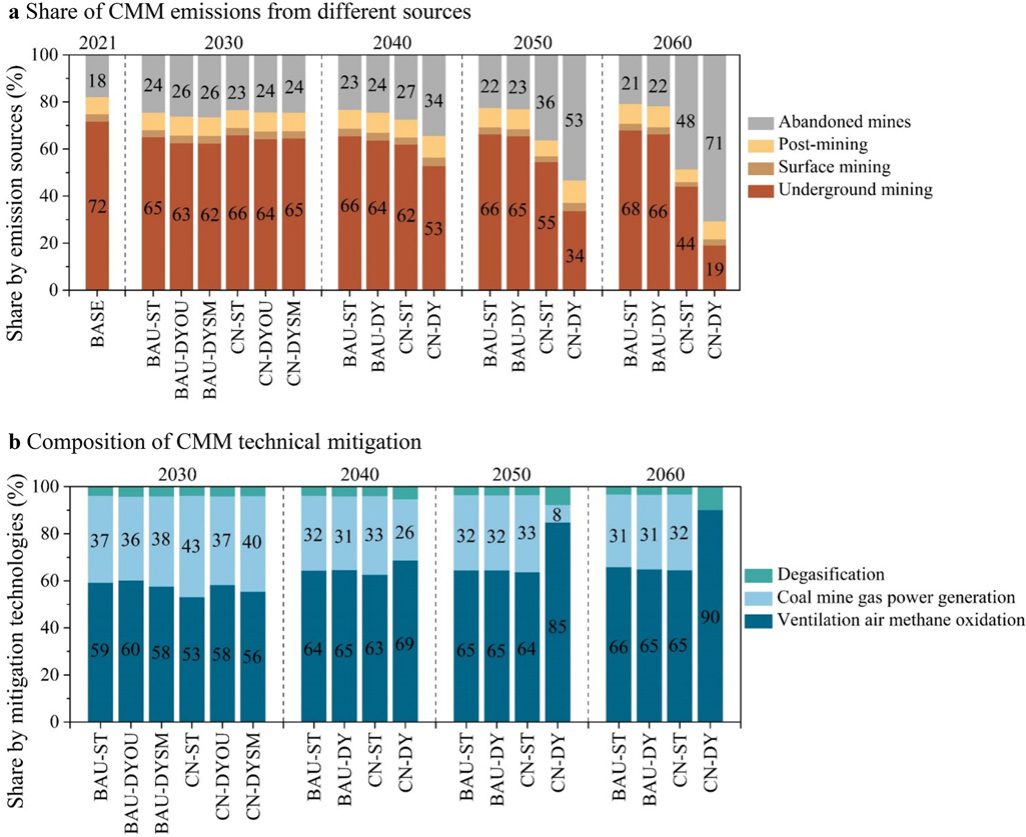
**Compositions of CMM emissions (a) and the mitigation potentials (b).** The scenarios are the same as in Fig. 2.

For different sources of CMM emissions (Fig. 3a), underground mining dominates the total emissions in the BAU scenario, accounting for over 60%. The share of emissions from surface mining and post mining fluctuates around 3% and 8%, respectively. In the CN scenario, the contribution of abandoned mines will rise sharply with the mass closure of coal mines, from 24% in 2030 to 71% in 2060. Abandoned coal mines overtake underground mining as the largest emission source of CMM.

As shown in Fig. 3b, the share of the three CMM mitigation technologies would follow the evolution of CH_4_ concentration. In the BAU scenario, more than 60% of the CMM technical mitigation comes from ventilation air methane oxidation technology that is suitable for low CH_4_-content coal mines. As the CH_4_ concentration continues to decrease in the CN scenario, this mitigation technology could play an increasing role in reducing CH_4_ emissions. Ventilation air methane oxidation contributes up to 90% of the CMM technical mitigation potential in CN-DY. In contrast, the lower share of this technology in the CN-ST scenario indicates its underrated contribution to CMM emission reductions.

#### 3.1.2 Evolution of regional coal mine methane emissions and mitigation potentials

**Fig. 4:**
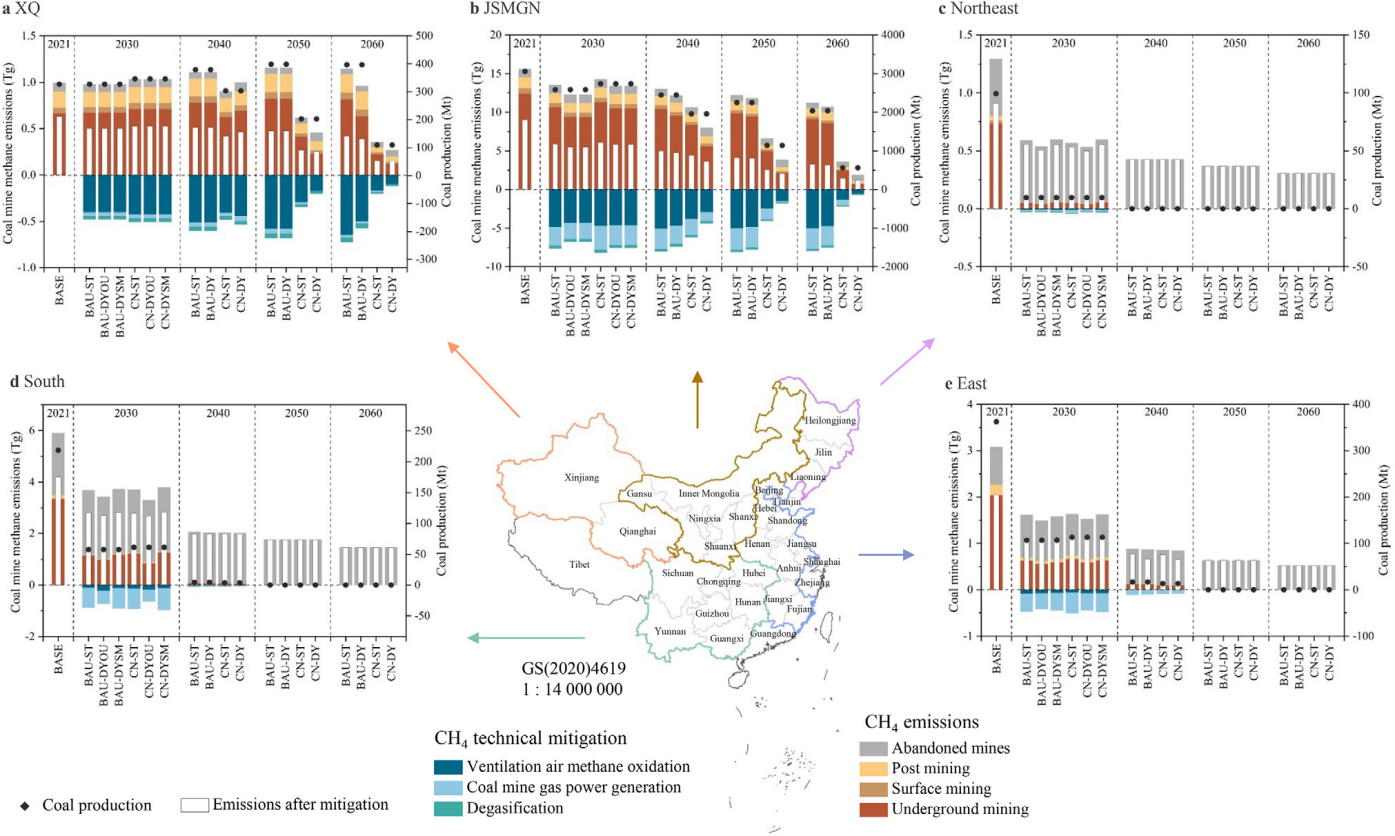
**CMM emissions and mitigation potentials in Chinese five coal regions from 2021 to 2060.** XQ (a) represents Xinjiang and Qinghai. JSMGN (b) represents Shanxi, Shaanxi, Inner Mongolia, Gansu and Ningxia. Northeast (c) represents Heilongjiang, Jilin, and Liaoning. South (d) represents Chongqing, Guangxi, Guizhou, Hubei, Hunan, Sichuan and Yunnan. East (e) represents Anhui, Beijing, Fujian, Hebei, Henan, Jiangsu, Jiangxi and Shandong. The scenarios are the same as in Fig. 2.

Following the division of the five coal production regions in China by the Chinese Academy of Engineering [35], we further explore the spatial distribution of CMM emissions and mitigation potential (Fig. 4). The XQ region (Xinjiang and Qinghai) was taken as a strategic coal reserve region for decades, while it contributed the smallest emissions in 2021, mainly owing to low EFs. This region is characterized by low CH_4_-content coal mines (over 90%), and average EFs are as low as the default EFs of post-underground mining. As a result, the share of post-mining in this region is the largest among the five regions. By 2030, Qinghai is expected to withdraw coal production. Xinjiang has abundant coal reserves and good mining conditions, and its coal production is expected to increase in the short term. The total CMM emissions in this region will grow slightly to 1.03 Tg in 2030 and drop rapidly to 0.27 Tg in 2060 under the CN-DY scenario. For technical mitigation, ventilation air methane oxidation will contribute approximately 85%, which would also be the highest share among all regions.

For the Northeast, South and East, their aggregated CMM emissions accounted for 38% of the domestic emissions in 2021, mainly from abandoned coal mines (Fig. 4c-e). As proposed in the 13th Five-Year Plan for the coal industry [36], coal mining will be compressed in the eastern area and restricted in the south and northeast. In 2030, CH_4_ emissions from abandoned coal mines in these regions would even exceed coal mining, especially in the northeast (approximately 90%). The technical mitigation potential will be lower than 1 Tg, and most of it will originate from coal-mine gas power generation. All regions have lower residual CMM emissions in 2030 under the CN-DYOU scenario than under the CN-DYSM scenario. Therein, the difference in residual emissions between the two scenarios in the southern region is the largest. The emission reduction advantages of closing outburst coal mines first are thus higher in the south than in other regions. In fact, these regions are characterized by high CH_4_-content underground coal mines, and there are no surface coal mines in the south and east. Furthermore, all coal mines in the northeastern region will be out of production by 2040 and in the south and east by 2050. Therefore, all of their CMM emissions would be from abandoned coal mines. The aggregated abandoned mine methane emissions of these three regions in the 2060 CN scenario will be 2.56 Tg, accounting for 85% of the national emissions from abandoned mines.

**Fig. 5:**
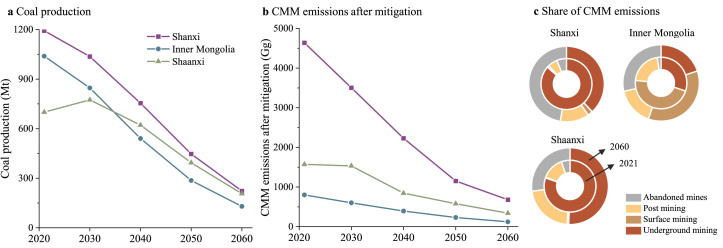
**Coal production (a), CMM emissions after mitigation (b), and composition of CMM emissions (c) in Shanxi, Inner Mongolia and Shaanxi under the carbon neutrality scenario**.

The JSMGN region (Shanxi, Shaanxi, Inner Mongolia, Gansu, and Ningxia) is the largest coal production and CMM emission region, contributing 75% of the domestic coal production and 58% of the total CMM emissions in 2021. The center of gravity of coal production would further shift to this region in the short and long term. In other words, despite the decline in coal production in this region, its contribution to total production would remain at about 84% during the period 2030–2060. As the three largest coal production bases in China, the aggregated coal production in Shanxi, Inner Mongolia and Shaanxi will reach 558.89 Mt in the CN-DY scenario (Fig. 5a). Shanxi and Inner Mongolia hold the top two positions ranked by coal production in the short term, yet Shaanxi takes Inner Mongolia's place during the period 2040–2060. In addition, Shaanxi, with less coal supply, produces more CMM emissions than Inner Mongolia during the accounting period. The JSMGN region will be the largest contributor to national CMM technical mitigation, accounting for more than 80%. The CMM emissions after mitigation in Shanxi, Shaanxi, and Inner Mongolia will be 678.47 Gg, 340.35 Gg, and 121.19 Gg in the CN-DY scenario, declining by 85%, 84% and 78% compared with the 2021 levels, respectively. The EF of underground coal mining in Inner Mongolia is the lowest, and the proportion of surface coal mines is the largest among all provinces. By 2060, 36% of CMM emissions in Inner Mongolia will originate from surface coal mines in the CN-DY scenario, even exceeding underground mines (Fig. 5c).

### 3.2 Cost analysis of coal mine methane mitigation

#### 3.2.1 National coal mine methane mitigation cost from 2021 to 2060

**Fig. 6:**
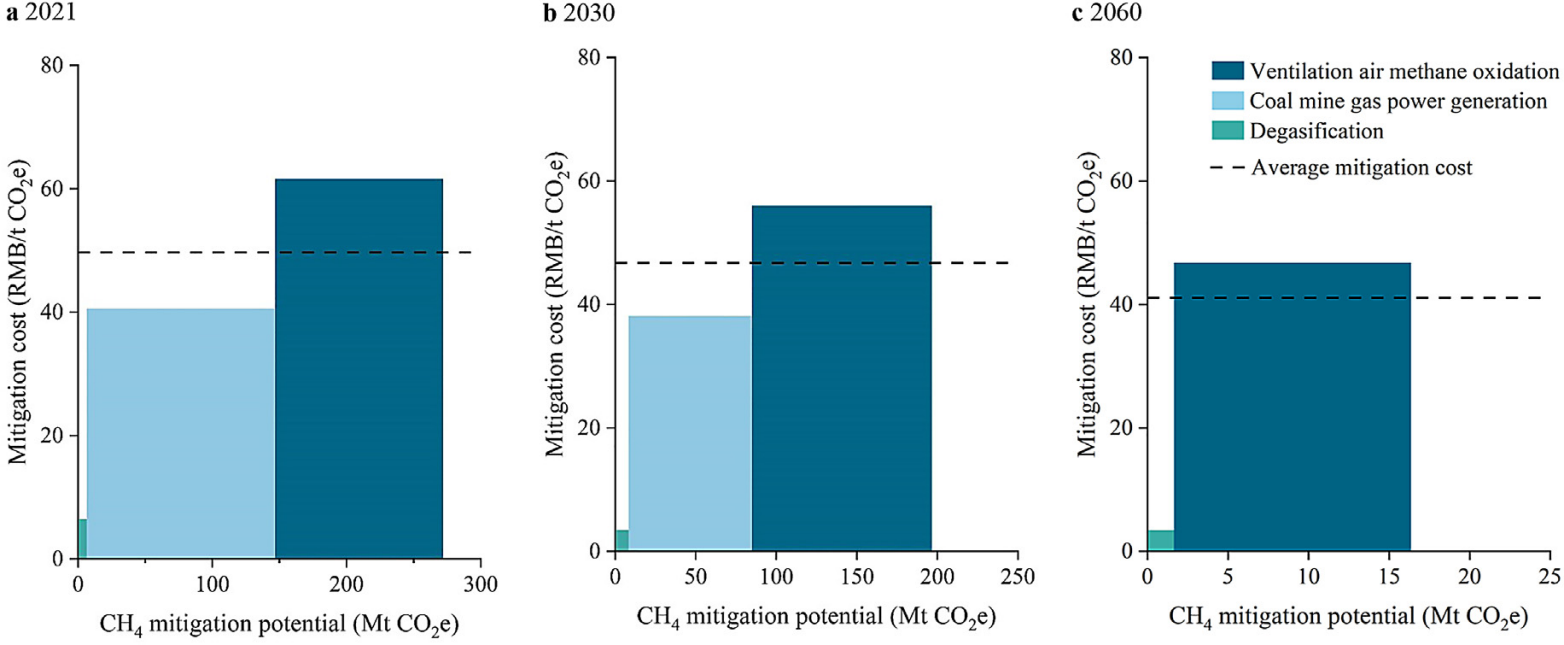
**The total abatement cost of China's CMM mitigation processes in 2021 (a), 2030 (b), and 2060 (c) under the carbon neutrality scenario.** The average mitigation cost is shown (y-axis). The widths of the bars represent the amount of mitigation potential (x-axis), and the area indicates the mitigation cost of the specific mitigation technology.

Currently, large-scale technical mitigation of CMM emissions is challenging, mainly because the CH_4_ concentration in CMM emissions is typically low and can fluctuate in quality and quantity. If the CMM mitigation technologies are deployed on a large scale in 2021, as shown in Fig. 6a, the average CMM mitigation cost would be approximately 1043.07 RMB/t CH_4_ (49.17 RMB/t CO_2_). The calculated CMM mitigation cost is converted into CO_2_-equivalent using the 100 yr global warming potential (the GWP of CH_4_ is 21) to compare with carbon prices. In 2021, the average carbon price in China's carbon market was 42.85 RMB/t CO_2_. Therefore, large-scale CMM technical mitigation is not economically feasible in 2021.

Considering technological advancements, we assume that it would be technically feasible to conduct large-scale CMM mitigation (except for abandoned coal mines) in the long run. Under such circumstances, the total mitigation cost of large-scale CMM technical mitigation in 2030 and 2060 will be 9.15 billion RMB and 0.69 billion RMB in the CN scenario, respectively. The average mitigation cost will decrease to 46.68 RMB/t CO_2_e in 2030 and 41.09 RMB/t CO_2_e in 2060. Based on the modeling scenario in GCAM toward carbon neutrality, the carbon price in 2030 is required to be 642.19 RMB/t. If the emission reduction price of CO_2_ could be close to the model simulation in the short term, then all three CMM mitigation technologies would be significantly cost-effective. Moreover, the carbon price will continue to increase for a carbon-neutral future. In such cases, the CMM emission reductions of 9.34 Tg in 2030 and 0.78 Tg in 2060 could thus be fully realized by continuing to strengthen policy measures and effectively guide the market.

#### 3.2.2 Provincial coal mine methane mitigation costs in 2030 and 2060

The average CMM mitigation costs of the Chinese coal-producing provinces in 2030 and 2060 are listed in Table 3. All provinces can achieve low-cost CMM mitigation (< 60 RMB/t CO_2_e) in the future. The average mitigation cost in 55% of coal-producing provinces in 2030 will be below 50 RMB/t CO_2_e, and this proportion will become 100% in 2060. Specifically, Inner Mongolia is the most cost-effective province for implementing technical CMM mitigation. The average CMM mitigation cost in Inner Mongolia is projected to be as low as 19.47 RMB/t CO_2_e by 2060. This can be explained by the large proportion of surface coal mines in Inner Mongolia. Meanwhile, this province has a higher CMM mitigation potential with coal production bases. Thus, priority should be given to investment in CMM mitigation in this province. In comparison, the average mitigation costs of other provinces with coal production bases (i.e., Shanxi, Shaanxi and Xinjiang) are much higher owing to the large proportion of low CH_4_-content underground mines. Additionally, it is also relatively easier to reduce CMM emissions in Anhui and Guizhou, which are characterized by high CH_4_-content underground coal mines, as the average mitigation costs are below 42 RMB/t CO_2_e in 2030.

**Table 3:** **Provincial average mitigation costs of CMM emissions under the carbon neutrality scenario (Unit: RMB/t CO**_**2**_**e)**.

	Ningxia	Shaanxi	Xinjiang	Shanxi	Inner Mongolia	
2030	55.04	52.74	50.46	47.45	21.86	
2060	46.04	46.17	4.40	44.64	19.47	
	Shandong	Henan	Gansu	Heilongjiang	Guizhou	Anhui
2030	52.10	50.60	46.77	45.39	41.53	38.53

## 4 Discussion and policy implications

We performed a prospective assessment of China's provincial CMM mitigation potentials and costs by integrating the dynamic EFs of CMM and the coal supply pathway. Dynamic EFs derived from point-source data can fully consider changes in coal mines (e.g., coal mine retirement); hence, this bottom-up evaluation is more accurate than previous research using static EFs. Our results also show that if the constant EFs of underground mines are used, the total emissions and mitigation will be overestimated. Compared with existing studies using the top-down method based on constant EFs, their projections are 27%−64% higher than that of this study (Table 4). In addition, based on the probability distribution of provincial EFs from various coal mines, we have estimated the uncertainties of CMM emissions in the baseline year using the Monte Carlo method (details in Supplementary Material). The results show that the overall uncertainty is in the range of −19% to 32%, which is narrower than the uncertainty estimates by China's official government inventory (−62%, 46%) [37], indicating higher confidence.

**Table 4:** **Comparison of CMM emissions and mitigation in 2030 between this study and previous research**.

	This study	Teng et al. [18]	Höglund-Isaksson [20]	USEPA[38]
Method	Bottom-up	Top-down	Top-down	Top-down
Total CMM emissions (Tg)	18.68–21.18	20.44–27.48	29.78	31.35
CMM technical mitigation potential (Tg)	4.99–8.11	7.03–13.31		

Our work demonstrates that by both reducing coal production and adopting CMM mitigation technologies, China's CMM emissions could fall by 35%−39% and 65%−78% from 2021 levels in 2030 and 2060, respectively. Given the absence of detailed guidance to reduce national CMM emissions, it is recommended to establish medium- and long-term CH_4_ mitigation goals in China's coal sector based on the above results. From the view of reducing coal supply, the shutdown of outburst coal mines rather than small mines should be a priority in the CMM mitigation strategy between 2021 and 2030, especially in the southern region. However, the long-term transition away from coal necessitates decommissioning all outburst coal mines, as well as small high and low CH_4_-content coal mines. For CMM mitigation technologies, ventilation air methane oxidation is predicted to account for the majority of CMM technical mitigation in the long run; therefore, there needs to be a greater focus on improving this technology. Moreover, regional CMM mitigation targets could be diversified to meet the overall national target. For example, 83% of baseline CMM emissions could be reduced in the JSMGN region with large coal production bases in 2060, whereas 60% could be reduced in the northeastern region with depleted coal resources.

It should be emphasized that as coal mines gradually become decommissioned, CH_4_ emission outbreaks from abandoned mines are imminent, implying the urgency of CH_4_ utilization in abandoned mines. Abandoned coal mines will overtake underground mining as the largest emission source of CMM in our carbon neutrality scenario (nearly 71%). Especially in the Northeast, South and East of China, abandoned coal mines may be the only source of CMM emissions in the coming decades. Given the long-term persistence of abandoned mine CH_4_ emissions, deep CH_4_ mitigation calls for specific policy measures for abandoned coal mine management in advance. Currently, owing to complex geological conditions and immature technologies, the overall resource utilization of abandoned mines in China is still in the experimental stage [39]. Thus, mitigation pilot projects for various types of abandoned coal mines are recommended to be set up as soon as possible, such as in the northeast, south and east. Particular attention needs to be paid to promoting continuous technological innovation for the utilization of abandoned mines.

We also found that although CMM technical mitigation is not currently cost-effective, it may be a feasible and suitable solution as CO_2_ trading prices increase. Furthermore, according to the Climate & Clean Air Coalition [40], the average mitigation costs of CH_4_ emissions in the oil and gas, agriculture, and waste treatment sectors are 153 RMB/t, 245 RMB/t, and 956 RMB/t CO_2_e, respectively. Consequently, the CMM mitigation cost would also be the lowest among all anthropogenic CH_4_ emitting sectors. The carbon trade market will play a vital role in future mitigation of overall CH_4_ emissions. Our results further suggest incorporating CMM mitigation into the carbon trade market to increase the enthusiasm of related enterprises for emission reduction. Enterprises or emission entities may gain revenue by participating in the emission trading market. As for the regional cost competitiveness of CMM mitigation, Inner Mongolia, with its higher mitigation potential and lower mitigation cost can be set as the priority in emission reduction strategies.

## 5 Conclusion

This study investigates China's CMM emissions, mitigation potentials, and related costs by 2060. We develop the methodology framework by combining dynamic EFs, updated data on abandoned coal mines and coal production forecasts. The results show that the CMM emissions will decrease to 3.68 Tg-5.88 Tg through reduced coal production and adoption of CMM abatement measures in 2060, which means a decline of 65%−78% compared with the 2021 level. The coal production bases (i.e., Shanxi, Inner Mongolia, Shaanxi, and Xinjiang) have the greatest CMM mitigation potentials. CH_4_ emissions from abandoned coal mines will remain significant through 2060, regardless of future coal production. In a carbon neutrality scenario, the contribution of abandoned coal mines to total emissions would reach 71%, and all CMM emissions in the northeast, south and east of China would solely come from abandoned coal mines. Additionally, the large-scale deployment of CMM mitigation technologies is currently challenging. However, it will be the most market-competitive in the long term when compared with carbon abatement and technical mitigation in other CH_4_-emitting sectors. The national CMM mitigation cost would be 41.09 RMB/t CO_2_e in the carbon neutrality scenario, and the mitigation cost of all coal-producing provinces will be below 50 RMB/t CO_2_e. Owing to the larger CMM mitigation potential and lower average mitigation cost, the priority of CMM technical mitigation should be given to Inner Mongolia, which is characterized by surface coal mines. These findings can be helpful in scientific China's CH_4_ mitigation target formulation to accelerate the process of carbon neutrality.

## Declaration of competing interest

The authors declare that they have no conflicts of interest in this work.

## Appendix Supplementary materials

Image, application 1
