# Supplementary material for: Methane mitigation potentials and related costs of China's coal mines
Source: Fundam Res. 2023 Dec 29;4(6):1688–95. doi: 10.1016/j.fmre.2023.09.012 (PMC11670681; doi:10.1016/j.fmre.2023.09.012)
Supplement: Supplementary file 1 [file mmc1.docx]

Supplementary Information for

**Methane mitigation potentials and related costs of China’s coal mines**

Yating Kang^a,1^, Peipei Tian^a,1^, Jiashuo Li^a,^*, Hetong Wang^a^, Kuishuang Feng^b,^*

^a^ Institute of Blue and Green Development, Shandong University, Weihai, 264209, P.R. China

^b^ Department of Geographical Sciences, University of Maryland, College Park, MD 20742, USA

* Corresponding author: lijiashuo@sdu.edu.cn (Jiashuo Li); kfeng@umd.edu (Kuishuang Feng)

^1^ These authors contributed equally to this work

**1. Weighted moving average (WMA) method for coal production preprocessing**

The historical provincial coal production data over the past decade are processed through the weighted moving average (WMA) method to better fit the production trend.

$\text{y}_{\text{i,j}}\text{=0.1×}\text{y}_{\text{i,j-2}}\text{+0.2×}\text{y}_{\text{i,j-1}}\text{+0.7×}\text{y}_{\text{i,j}}\text{ (2011≤i≤2021)}$ (1)

where *y_i,j_* is the weighted averaged coal production of province *i* for year *j*; 0.1, 0.2, and 0.7 are assigned weights; *y_i,j-2_*, *y_i,j-1_*, *y_i,j_* were the actual coal production of province *i* for year *i-2*, year *i-1*, and year *i*, respectively.

The initial forecasted values of coal production based on the preprocessed data are presented in Table S1, and the final adjusted values in Table S2. To measure the prediction accuracy of our forecasting method, the mean absolute percentage error (MAPE) of forecasted values was subsequently used to validate. MAPE is less than 10%, meaning excellent forecasting and 10-20% for good forecasting.

**Table S1**. Fitted (2011-2021) and initial forecasted (2030-2060) coal production in China (Unit: million tons)

| Province | Fitted values | | | | | | | | | MAPE | Initial forecasted values | | | |
| --- | --- | --- | --- | --- | --- | --- | --- | --- | --- | --- | --- | --- | --- | --- |
|  | 2013 | 2014 | 2015 | 2016 | 2017 | 2018 | 2019 | 2020 | 2021 |  | 2030 | 2040 | 2050 | 2060 |
| Anhui | 139.70 | 135.46 | 131.22 | 126.98 | 122.74 | 118.50 | 114.26 | 110.02 | 105.79 | 7.31 | 67.64 | 25.25 | - | - |
| Chongqing | 39.27 | 34.73 | 30.20 | 25.66 | 21.12 | 16.58 | 12.05 | 7.51 | 2.97 | - | - | - | - | - |
| Fujian | 19.40 | 17.57 | 15.74 | 13.91 | 12.08 | 10.25 | 8.42 | 6.59 | 4.76 | 10.84 | - | - | - | - |
| Gansu | 46.77 | 45.67 | 44.58 | 43.48 | 42.38 | 41.28 | 40.19 | 39.09 | 37.99 | 6.98 | 28.11 | 17.14 | 6.16 | - |
| Guangxi | 6.91 | 6.35 | 5.79 | 5.24 | 4.68 | 4.12 | 3.57 | 3.01 | 2.45 | 17.57 | - | - | - | - |
| Guizhou | 182.82 | 176.31 | 169.80 | 163.29 | 156.77 | 150.26 | 143.75 | 137.24 | 130.73 | 8.23 | 72.12 | 7.00 | - | - |
| Hebei | 91.39 | 85.25 | 79.12 | 72.99 | 66.85 | 60.72 | 54.59 | 48.45 | 42.32 | 15.62 | - | - | - | - |
| Heilongjiang | 80.56 | 76.53 | 72.50 | 68.47 | 64.45 | 60.42 | 56.39 | 52.36 | 48.33 | 15.60 | 12.08 | - | - | - |
| Henan | 159.72 | 151.85 | 143.99 | 136.12 | 128.26 | 120.39 | 112.53 | 104.66 | 96.80 | 9.98 | 26.01 | - | - | - |
| Hubei | 10.47 | 9.06 | 7.65 | 6.24 | 4.82 | 3.41 | 2.00 | 0.59 | 0.01 | - | - | - | - | - |
| Hunan | 69.56 | 60.44 | 51.32 | 42.20 | 33.09 | 23.97 | 14.85 | 5.73 | -3.39 | 17.24 | - | - | - | - |
| Inner Mongolia | 960.39 | 962.56 | 964.73 | 966.90 | 969.06 | 971.23 | 973.40 | 975.57 | 977.74 | 13.21 | 997.27 | 1018.97 | 1040.66 | 1062.36 |
| Jiangsu | 20.45 | 19.00 | 17.55 | 16.10 | 14.64 | 13.19 | 11.74 | 10.29 | 8.83 | 10.71 | - | - | - | - |
| Jiangxi | 29.30 | 25.57 | 21.84 | 18.11 | 14.38 | 10.66 | 6.93 | 3.20 | -0.53 | 18.03 | - | - | - | - |
| Jilin | 41.77 | 36.94 | 32.11 | 27.28 | 22.46 | 17.63 | 12.80 | 7.97 | 3.15 | 14.30 | - | - | - | - |
| Liaoning | 58.75 | 54.41 | 50.07 | 45.72 | 41.38 | 37.04 | 32.69 | 28.35 | 24.01 | 15.59 | - | - | - | - |
| Ningxia | 82.74 | 81.88 | 81.01 | 80.14 | 79.28 | 78.41 | 77.54 | 76.68 | 75.81 | 9.05 | 68.02 | 59.36 | 50.70 | 42.04 |
| Qinghai | 21.26 | 19.28 | 17.30 | 15.31 | 13.33 | 11.34 | 9.36 | 7.38 | 5.39 | 15.07 | - | - | - | - |
| Shaanxi | 471.58 | 497.51 | 523.44 | 549.37 | 575.29 | 601.22 | 627.15 | 653.08 | 679.01 | 7.77 | 912.36 | 1171.64 | 1430.91 | 1690.19 |
| Shandong | 159.28 | 152.23 | 145.18 | 138.13 | 131.07 | 124.02 | 116.97 | 109.92 | 102.87 | 9.25 | 39.40 | - | - | - |
| Shanxi | 878.48 | 898.62 | 918.77 | 938.91 | 959.05 | 979.19 | 999.34 | 1019.48 | 1039.62 | 12.10 | 1220.91 | 1422.34 | 1623.77 | 1825.20 |
| Sichuan | 82.79 | 74.93 | 67.06 | 59.19 | 51.33 | 43.46 | 35.59 | 27.73 | 19.86 | 14.88 | - | - | - | - |
| Xinjiang | 129.52 | 145.91 | 162.30 | 178.69 | 195.09 | 211.48 | 227.87 | 244.26 | 260.65 | 14.89 | 408.18 | 572.10 | 736.03 | 899.95 |
| Yunnan | 81.82 | 76.27 | 70.73 | 65.18 | 59.64 | 54.09 | 48.55 | 43.00 | 37.46 | 13.83 | - | - | - | - |

**Table S2**. Final projections of provincial coal production in China (Unit: million tons)

| Province | Business as usual scenario | | | | Carbon neutrality scenario | | | |
| --- | --- | --- | --- | --- | --- | --- | --- | --- |
|  | 2030 | 2040 | 2050 | 2060 | 2030 | 2040 | 2050 | 2060 |
| Anhui | 54.21 | 16.74 | - | - | 57.41 | 13.39 | - | - |
| Gansu | 22.53 | 11.36 | 3.34 | - | 23.86 | 9.09 | 1.70 | - |
| Guizhou | 57.80 | 4.64 | - | - | 61.21 | 3.71 | - | - |
| Heilongjiang | 9.68 | - | - | - | 10.26 | - | - | - |
| Henan | 20.85 | - | - | - | 22.08 | - | - | - |
| Inner Mongolia | 799.26 | 675.58 | 564.02 | 468.82 | 846.48 | 540.45 | 286.52 | 129.70 |
| Ningxia | 54.51 | 39.35 | 27.48 | 18.55 | 57.73 | 31.48 | 13.96 | 5.13 |
| Shaanxi | 731.20 | 776.80 | 775.53 | 745.89 | 774.41 | 621.42 | 393.96 | 206.36 |
| Shandong | 31.57 | - | - | - | 33.44 | - | - | - |
| Shanxi | 978.49 | 943.02 | 880.05 | 805.47 | 1036.31 | 754.39 | 447.06 | 222.84 |
| Xinjiang | 327.14 | 379.31 | 398.91 | 397.15 | 346.47 | 303.44 | 202.64 | 109.87 |

**Table S3**. The coal production from surface mining and its share of total production

|  | Shanxi | Inner Mongolia | Liaoning | Heilongjiang | Yunnan | Shaanxi | Qinghai | Ningxia | Xinjiang |
| --- | --- | --- | --- | --- | --- | --- | --- | --- | --- |
| Coal production (Mt) | 137 | 655 | 7 | 26 | 28 | 16 | 4 | 2 | 71 |
| Share (%) | 12.4 | 68 | 23.9 | 47.5 | 52.7 | 2.4 | 36.8 | 2.5 | 22.9 |

Note: The coal production from surface mining in provinces not listed in the table have not been recorded in any reports and literature, so we assume that total coal production in these provinces is from underground mining.

**Table S4**. The number of abandoned coal mines in China by 2021

|  | 2010-2019 | 2020 | 2021 | Total |
| --- | --- | --- | --- | --- |
| Hebei | 332 | 10 | 0 | 342 |
| Shanxi | 1758 | 32 | 0 | 1790 |
| Inner Mongolia | 73 | 0 | 6 | 79 |
| Liaoning | 312 | 2 | 0 | 314 |
| Jilin | 167 | 0 | 0 | 167 |
| Heilongjiang | 820 | 8 | 0 | 828 |
| Jiangsu | 21 | 0 | 0 | 21 |
| Anhui | 140 | 0 | 0 | 140 |
| Fujian | 382 | 0 | 0 | 382 |
| Jiangxi | 452 | 23 | 2 | 477 |
| Shandong | 127 | 7 | 0 | 134 |
| Henan | 625 | 6 | 15 | 646 |
| Hubei | 398 | 0 | 0 | 398 |
| Hunan | 1390 | 32 | 23 | 1445 |
| Guangxi | 85 | 2 | 1 | 88 |
| Chongqing | 816 | 0 | 14 | 830 |
| Sichuan | 964 | 50 | 5 | 1019 |
| Guizhou | 959 | 172 | 51 | 1182 |
| Yunnan | 1423 | 54 | 0 | 1477 |
| Shaanxi | 427 | 20 | 0 | 447 |
| Gansu | 274 | 10 | 0 | 284 |
| Qinghai | 44 | 0 | 0 | 44 |
| Ningxia | 63 | 0 | 0 | 63 |
| Xinjiang | 199 | 0 | 0 | 199 |

**Table S5**. Emission factors of underground coal mining in China (Unit: kg CH_4_/t coal)

| Province |  | Business as usual scenario | | | | | Carbon neutrality scenario | | | | |
| --- | --- | --- | --- | --- | --- | --- | --- | --- | --- | --- | --- |
|  | 2021 | 2030  -OU | 2030  -SM | 2040 | 2050 | 2060 | 2030  -OU | 2030  -SM | 2040 | 2050 | 2060 |
| Shanxi | 8.914 | 7.591 | 7.591 | 7.976 | 8.496 | 8.607 | 8.175 | 8.175 | 6.828 | 3.813 | 1.970 |
| Inner Mongolia | 1.105 | 0.958 | 0.958 | 0.958 | 0.958 | 0.958 | 0.958 | 0.958 | 0.958 | 0.958 | 0.958 |
| Heilongjiang | 10.178 | 8.551 | 11.250 | - | - | - | 8.508 | 11.250 | - | - | - |
| Anhui | 8.030 | 7.927 | 7.927 | 7.565 | - | - | 7.942 | 7.942 | 7.611 | - | - |
| Shandong | 1.281 | 1.219 | 1.219 | - | - | - | 1.219 | 1.219 | - | - | - |
| Henan | 7.571 | 4.385 | 6.275 | - | - | - | 4.385 | 6.466 | - | - | - |
| Guizhou | 20.161 | 17.242 | 20.771 | 15.410 | - | - | 14.141 | 20.883 | 15.745 | - | - |
| Shaanxi | 3.611 | 3.611 | 3.611 | 3.611 | 3.576 | 3.280 | 3.611 | 3.611 | 1.474 | 1.474 | 1.474 |
| Gansu | 2.851 | 1.628 | 1.628 | 1.628 | 1.628 | - | 1.628 | 1.628 | 1.628 | 1.628 | - |
| Ningxia | 2.522 | 1.146 | 1.146 | 1.146 | 1.146 | 1.146 | 1.146 | 1.146 | 1.146 | 1.146 | 1.146 |
| Xinjiang | 2.673 | 2.673 | 2.673 | 2.673 | 2.673 | 2.072 | 2.673 | 2.673 | 2.979 | 1.474 | 1.474 |

Note: OU refers to strategies of early retirement of outburst coal mines. SM refers to strategies of early retirement of small coal mines.

**Table S6**. The default emission factors of abandoned coal mines (million m^3^/mine) [1]

| Interval of mine closure | Inventory Year | | | | |
| --- | --- | --- | --- | --- | --- |
|  | 2021 | 2030 | 2040 | 2050 | 2060 |
| 2021-2025 | 0.555 | 0.439 | 0.382 | 0.343 | 0.301 |
| 2026-2060 | - | 1.265 | 0.675 | 0.507 | 0.317 |

**Table S7**. Cost factors and removal efficiency improvements of CMM mitigation technologies [2]

|  | Nascent technology | | Mature technology | |
| --- | --- | --- | --- | --- |
|  | Cost factor | Removal efficiency improvement | Cost factor | Removal efficiency improvement |
| 2030 | 0.91 | 1.1 | 0.94 | 1.05 |
| 2040 | 0.85 | 1.2 | 0.9 | 1.1 |
| 2050 | 0.8 | 1.3 | 0.87 | 1.15 |
| 2060 | 0.76 | 1.4 | 0.84 | 1.2 |

Note: Ventilation air methane oxidation is nascent technology, degasification and coal mine gas power generation are mature technologies.

**Table S8**. Uncertainties of CMM emissions in 2021 measured using the Monte Carlo method (Unit: Gg)

|  | Mean | 95%CI | |
| --- | --- | --- | --- |
|  |  | Lower | Upper |
| Shanxi | 6998.2 | 4590.81 | 9613.48 |
| Inner Mongolia | 1138.45 | 867.87 | 1615.62 |
| Liaoning | 116.4 | 79.17 | 164.14 |
| Jilin | 67.26 | 43.31 | 98.32 |
| Heilongjiang | 251.9 | 218.89 | 304.37 |
| Jiangsu | 29.25 | 24.27 | 34.71 |
| Anhui | 818.87 | 551.28 | 1037.45 |
| Fujian | 22.28 | 20.75 | 30.65 |
| Jiangxi | 45.07 | 41.7 | 62.52 |
| Shandong | 87.28 | 68.52 | 134.23 |
| Henan | 641.97 | 586.46 | 875.4 |
| Hubei | 1.66 | 1.51 | 2.29 |
| Hunan | 144.07 | 100.98 | 210.21 |
| Guangxi | 16.48 | 11.69 | 21.2 |
| Chongqing | 201.79 | 184.61 | 274.28 |
| Sichuan | 469.04 | 384.08 | 585.44 |
| Guizhou | 2436.17 | 1908.93 | 3077.28 |
| Yunnan | 353.94 | 327.02 | 471.65 |
| Shaanxi | 2506.47 | 2008.51 | 3092.3 |
| Gansu | 60.39 | 55.42 | 82.7 |
| Qinghai | 7.6 | 6.97 | 9.64 |
| Ningxia | 236.12 | 196.85 | 303.12 |
| Xinjiang | 723.18 | 668.05 | 961.34 |
| Total | 17635.5 | 14312.69 | 23347.18 |


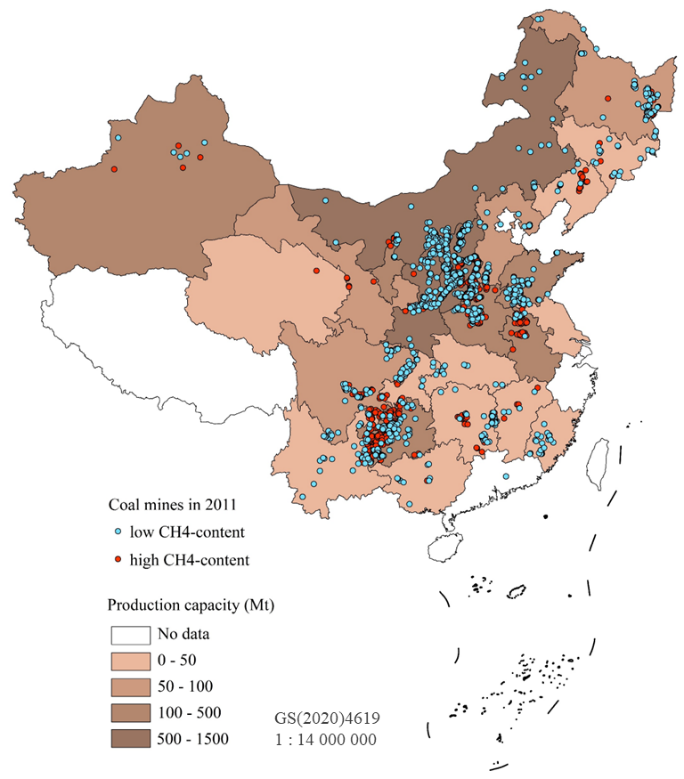


**Fig. S1.** Spatial distribution of coal mines in China in 2021.

**References**

[1] Intergovernmental Panel on Climate Change (IPCC), Refinement to the 2006 IPCC Guidelines for National Greenhouse Gas Inventories, 2019.

[2] U.S. Environmental Protection Agency (USEPA), Global Non-CO2 Greenhouse Gas Emission Projections & Mitigation 2015-2050, 2019.
